# Causal association between systemic lupus erythematosus and the risk of dementia: A Mendelian randomization study

**DOI:** 10.3389/fimmu.2022.1063110

**Published:** 2022-12-08

**Authors:** Tianyu Jin, Wei Huang, Fangzheng Cao, Xinyue Yu, Shunyuan Guo, Zhenhua Ying, Chao Xu

**Affiliations:** ^1^ Center for Rehabilitation Medicine, Department of Neurology, Zhejiang Provincial People’s Hospital, Affiliated People’s Hospital, Hangzhou Medical College, Hangzhou, China; ^2^ The Second Clinical Medical College, Zhejiang Chinese Medical University, Hangzhou, China; ^3^ Rheumatism and Immunity Research Institute, Zhejiang Provincial People’s Hospital, Affiliated People’s Hospital, Hangzhou Medical College, Hangzhou, China; ^4^ Alberta institute, Wenzhou Medical University, Wenzhou, China

**Keywords:** systemic lupus erythematosus, dementia, causality, Mendelian randomization, risk

## Abstract

**Introduction:**

It is well-documented that systemic lupus erythematosus (SLE) is associated with dementia. However, the genetic causality of this association remains unclear. Mendelian randomization (MR) was used to investigate the potential causal relationship between SLE and dementia risk in the current study.

**Methods:**

We selected 45 single nucleotide polymorphisms (SNPs) associated with SLE from publicly available genome-wide association studies (GWAS). Summary level statistics were obtained from the dementia GWAS database. MR estimates were performed using the inverse variance weighted (IVW) method, MR-Egger method and weighted median (WM) method. Cochran’s Q test, the intercept of MR-Egger, MR-Pleiotropy Residual Sum and Outlier method, leave-one-out analysis and funnel plot were applied for sensitivity analyses.

**Results:**

No significant causal association was found between SLE and any type of dementia, including Alzheimer’s disease, vascular dementia, frontotemporal dementia, and dementia with Lewy bodies. These findings were robust across several sensitivity analyses.

**Conclusion:**

Overall, our findings do not support a causal association between SLE and dementia risk.

## Introduction

Dementia is a common neurodegenerative disease with clinical manifestations as a severe decline in cognitive function leading to disruptions in family, occupational and daily life (1). The worldwide prevalence of dementia is estimated to be as high as 7% in the population over age 65 (2). This undoubtedly imposes an immense financial and healthcare burden on individuals, families, medical institutions and society. Alzheimer’s disease (AD) is the most common type of dementia, which accounts for approximately 50%-70% of dementia cases. Other common types of dementia include dementia with Lewy bodies (DLB), vascular dementia (VaD), frontotemporal dementia (FTD), and mixed dementia (3, 4). It is well-accepted that the interaction of advanced age, genetic factors, environmental triggers, and metabolic disorders contribute to the initiation and development of dementia (5, 6).

Systemic lupus erythematosus (SLE) is a chronic, systemic autoimmune disease characterized by autoantibody production and multisystem inflammation, predominantly affecting women of childbearing age (7). In recent decades, the prevalence ranges from 20 to 150 cases per 100,000 population and has been increasing yearly (8). SLE has wide clinical heterogeneity and is defined as neuropsychiatric lupus (NPSLE) when it is associated with neurological and psychiatric symptoms (9). The American College of Rheumatology (ACR) defined nineteen NPSLE syndromes in the late 20^th^ century, such as seizures, cerebrovascular disease, anxiety disorders, movement disorders and cognitive dysfunction. Of these, cognitive impairment is the most common which comprises one or more clinical manifestations, such as decreased attention, memory loss, and word-finding difficulties (10–12). This is similar to the American Psychiatric Association (APA) definition of dementia (13). A meta-analysis involving 11 observational studies reported a significantly increased risk of dementia in SLE patients (14). However, owing to the potential biases from residual confounding and the possibility of reverse causality, the genetic causality of this association remains unclear.

Indeed, previous epidemiological studies have shown powerful associations between a variety of risk factors and disease, whereas subsequent studies have demonstrated that these associations are due to interference from residual confounding factors rather than causal associations. Some typical examples include associations between vitamin E and atherosclerotic cardiovascular disease (15, 16), and β-carotene and lung cancer (17, 18). With the recent increased availability of genome-wide association studies (GWAS) databases, mendelian randomization (MR) research has received much attention. The evidence level of the MR studies sits at the interface of randomized controlled trials (RCTs) and observational studies (19), it can mimic an RCT and promise to be a robust statistical approach using instrumental variables (IVs) to clarify the causal association between exposure factors and disease (20). Causality in conventional observational studies is susceptible to interference by potential confounding factors and reverse causality. In MR analysis, alleles are randomly assigned from parents to offspring based on Mendel’s law of inheritance (21). Therefore, offspring genotypes are hardly associated with confounding factors. Additionally, MR analysis was able to avoid the problem of reverse causality since genotypes precede exposure in time (22, 23).

In the present study, we performed a two-sample MR analysis using the GWAS database to examine the genetic causality between SLE and common types of dementia risk.

## Methods

### Study design

We used the publicly available GWAS catalog to conduct a two-sample MR study. No additional ethical approval was required due to the re-analysis of previously summary-level data. Two-sample MR (version 0.5.5) and R (version 4.2.1) were used for MR analysis.

**Figure 1:**
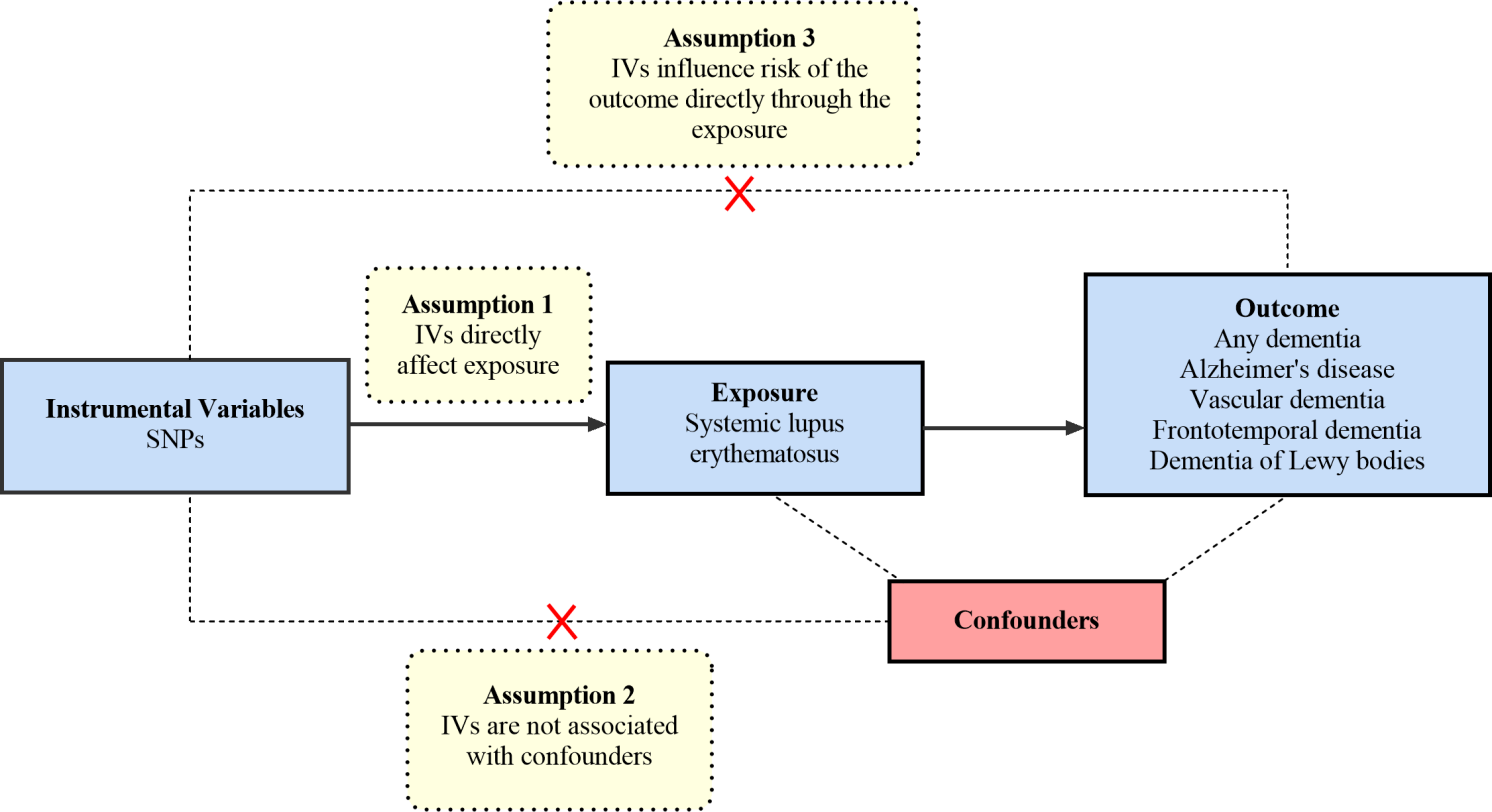
An overview of the study design. SNP, single nucleotide polymorphisms.

The MR analysis is based on the following three core hypotheses: 1) The selected IVs must be significantly associated with exposure (SLE) (24). We calculate the F-statistic to assess the strength of each genetic instrument. The following formula determines the F-statistic: F=R^2^×(N − 2)/(1 − R^2^); R^2^ = 2×EAF×(1−EAF)×β^2^ (25). In this formula, R^2^ refers to the cumulative explained variance of the selected IVs on SLE and EAF refers to the effect allele frequency, β refers to the estimated effect of SNP, and N refers to the sample size of the GWAS. If the F-statistic is greater than 10, the IV has a strong potential to predict dementia. 2) The selected IVs are not allowed to affect the outcome (dementia) through other pathways, only through specified exposure (SLE) (26). 3) Confounding factors are not associated with the selected IVs. The overview of the research design is shown in **Figure 1**.

### GWAS data for systemic lupus erythematosus

We extracted single nucleotide polymorphisms (SNPs) from the GWAS database as genetic IVs (24). The significant SNPs associated with SLE (*P*<5×10^-8^) were obtained from the latest and most extensive GWAS database, including 14,267 individuals of European ancestry (5,201 cases and 9,066 controls) (27) (**Table 1**). In order to avoid the potential bias caused by strong linkage disequilibrium (LD), we selected SNPs with LDr^2^ < 0.001.

**Table 1:** Details of the GWAS included in the Mendelian randomization.

Year	Trait	Population	Cases	Controls	Samplesize	Websource
2015	Systemic lupus erythematosus	European	5,201	9,066	14,267	DOI: 10.1038/ng.3434
2021	Any dementia	European	7,284	209,487	216,771	www.finngen.fi/en
2022	Alzheimer's disease	European	954	487,331	488,285	DOI: 10.3390/nu14091697
2021	Vascular dementia	European	881	211,508	212,389	www.finngen.fi/en
2010	Frontotemporal dementia	European	515	2,509	3,024	DOI: 10.1038/ng.536
2021	Dementia with Lewy bodies	European	2,591	4,027	6,618	DOI: 10.1038/s41588-021-00785-3

GWAS, Genome-Wide Association Studies.

### GWAS data for dementia

GWAS summary data for AD were obtained from an MR study with 954 cases and 487,331 control from the population of European ancestry (28). Summary-level GWAS data with VaD were extracted from the Finn consortium, including 212,389 participants of European ancestry (881 cases and 211,508 controls). Summary statistics for FTD from an international multicenter study comprising 515 cases and 2,509 controls of European ancestry (29). The GWAS data for DLB were derived from another independent GWAS multicenter study with a total of 2,591 cases and 4,027 controls (30). The GWAS summary data in our study are detailed in **Table 1**.

### Statistical analysis

MR estimates of SLE for the risk of dementia were calculated using the inverse variance weighting (IVW) method, weighted median (WM) method and MR-Egger method. The IVW method is the major MR analysis in our study, and it applies a meta-analysis method to integrate the Wald ratio of individual SNPs, which can be assumed that IVs can only influence outcomes through specified exposure. If there is no horizontal pleiotropy, the IVW method is able to achieve unbiased causal estimates (31). Therefore, the IVW method provides the most accurate assessment (32). The WM method and MR-Egger method were applied to the complement of analysis to investigate the bias due to ineffective IV and horizontal pleiotropy effects (33). The estimates of the MR-Egger method are probably inaccurate due to the influence of outlying genetic variants (34). The WM method has a relatively small bias, while its precision is lower, particularly the percentage of IVs with horizontal pleiotropy < 50% (35).

Sensitivity analysis is essential to evaluate potential heterogeneity and horizontal pleiotropy. Cochran’s Q test was performed to assess the heterogeneity of effect sizes for selected genetic IVs. The MR-Pleiotropy Residual Sum and Outlier method (MR-PRESSO) analysis was also applied to exclude outliers and moderate horizontal pleiotropy (35). The intercept derived from MR-Egger regression was employed to evaluate vertical pleiotropy (36). Leave-one-out analysis was conducted to explore the effect of removing one of the selected individual SNPs on the overall results (37).

### Process of MR analysis

**Figure 2:**
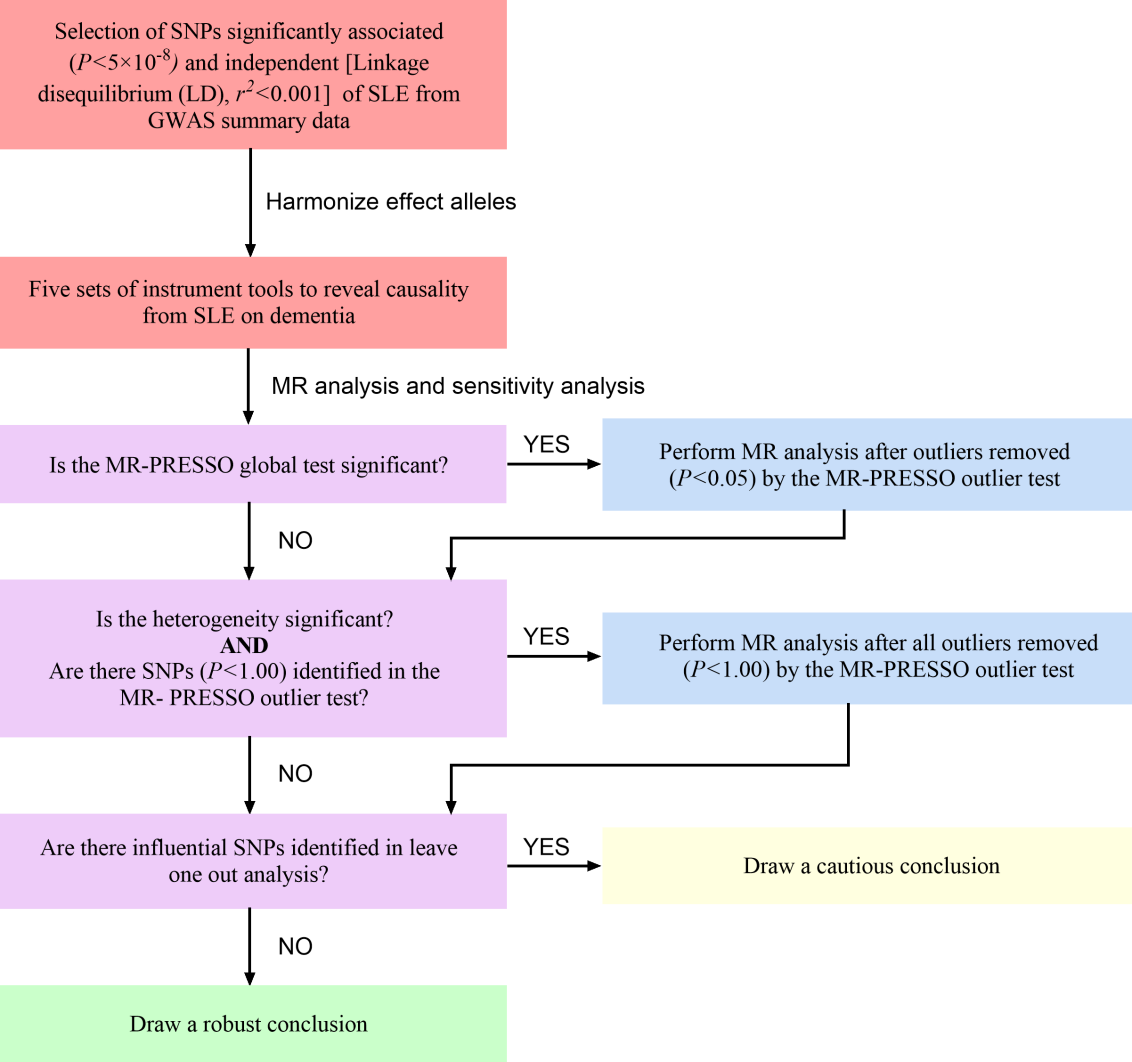
The flow chart of the MR process. SLE, systemic lupus erythematosus; SNP, single nucleotide polymorphisms; MR-PRESSO, MR-Pleiotropy Residual Sum and Outlier.

Our MR research was conducted according to the guideline of the STROBE-MR Statement (38). The flow chart of the MR process is shown in **Figure 2**.

We first harmonized the above-selected SNPs with effect allele in the database of dementia (all dementia, AD, VaD, FTD and DLB). Five sets of genetic instruments were finally extracted to clarify the genetic causality between SLE and dementia. Subsequently, we conduct the MR-PRESSO analysis to moderate horizontal pleiotropy. If the global test *P* value <0.05, which suggests significant horizontal pleiotropy in MR analysis, we will remove SNPs with *P* value <0.05 in the MR-PRESSO outlier test and re-perform the MR analysis. If the heterogeneity remains significant, we will remove all the outliers (*P*<1.00). Finally, we can draw a solid conclusion if the leave-one-out analysis fails to detect SNPs that potentially affect the stability of the outcomes (37).

## Results

### Genetic instruments for systemic lupus erythematosus

We finally included 45 significant (*P*<5×10^-8^) and independent (LDr^2^<0.001) SNPs as genetic instrumental variables, all of which had an F-statistic > 80, indicating no weak instrumental bias. The detailed information on 45 SNPs is illustrated in **Table S1**. Finally, the summary information of SNPs for SLE and dementia is presented in **Tables S2.1** -**S2.5**.

### Causal effect from systemic lupus erythematosus to dementia

**Figure 3:**
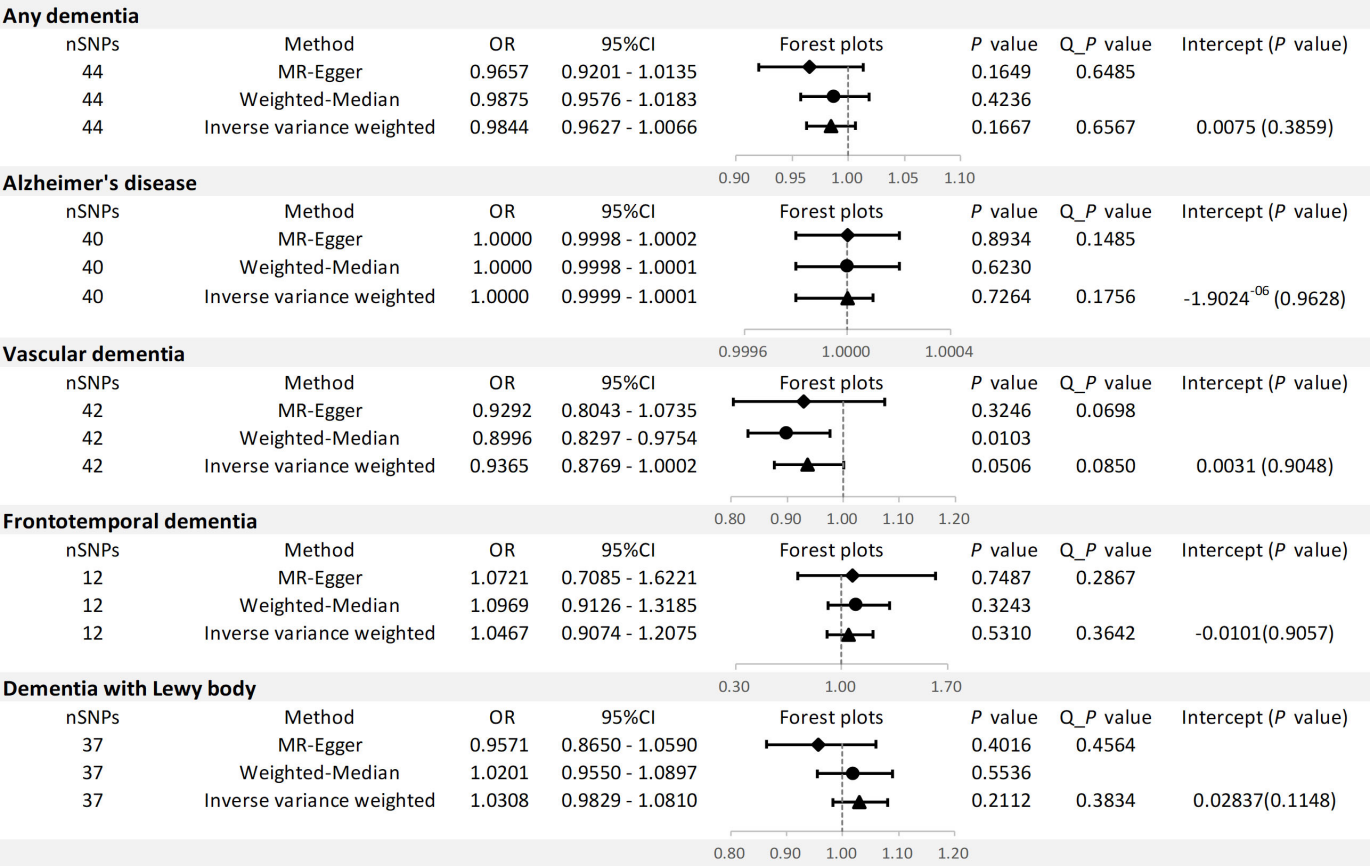
MR results and sensitivity analysis for association of SLE and dementia risk.

The results of the MR analysis are shown in **Figure 3**.

**Figure 4:**
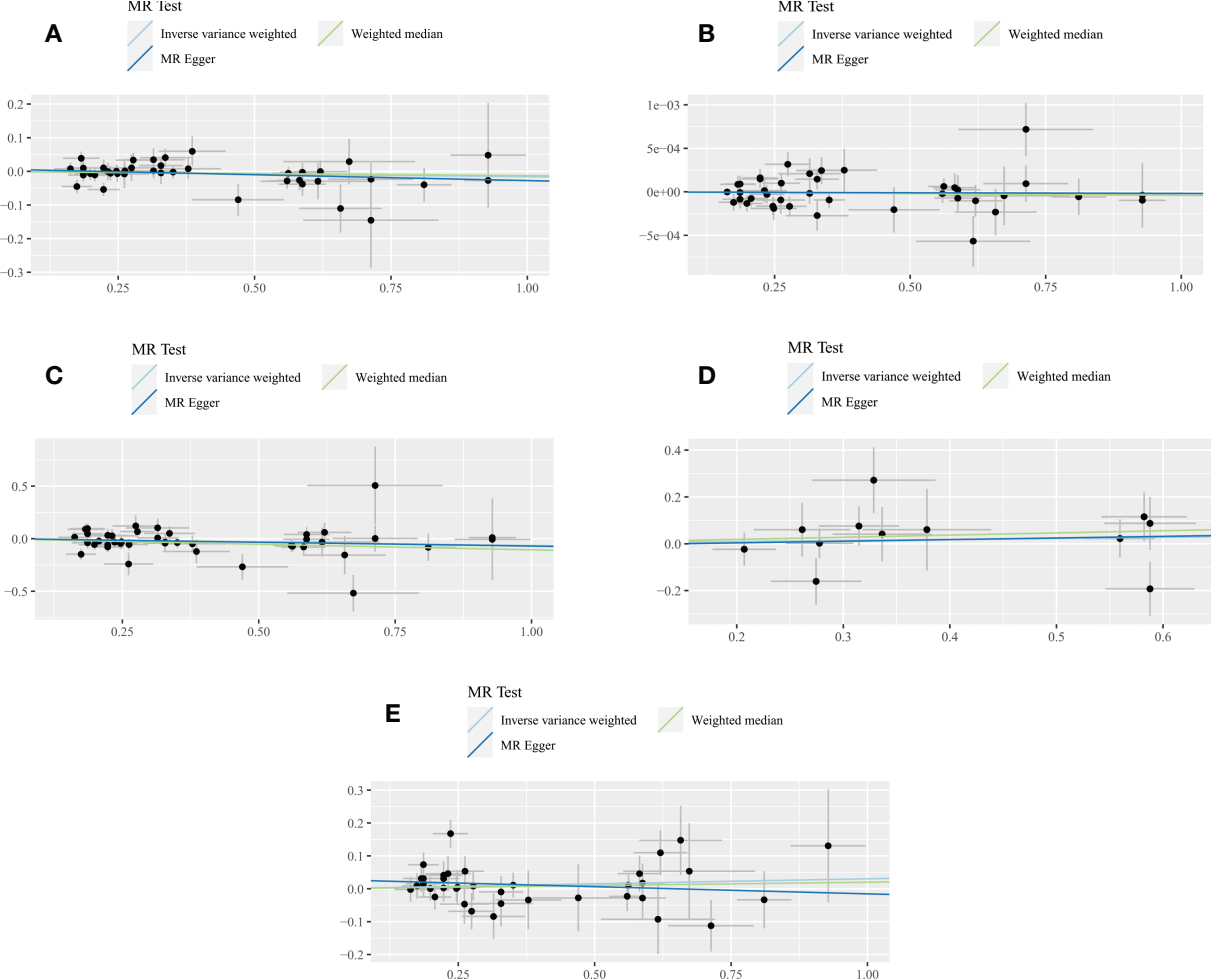
Scatter plot of the association between SLE and all dementia **(A)**, Alzheimer’s disease **(B)**, vascular dementia **(C)**, frontotemporal dementia **(D)**, dementia with lewy body **(E)**. Three lines reveal the estimated effect sizes by MR methods (inverse‐variance weighted, MR-Egger and weighted median).

For any dementia, no significant causal relationship was found for SLE and risk of any dementia [odds ratio (OR)=0.9884, 95% confidence interval (CI): 0.9627-1.0066, *P*=0.1667], this finding was similar to MR-Egger (OR=0.9657, 95% CI: 0.9201-1.0135, *P*=0.1649) and WM (OR=0.9875, 95% CI: 0.9576-1.0183, *P*=0.4236) (**Figure 4A**). No significant heterogeneity (Cochran’s Q *P*=0.6567) and horizontal pleiotropy (*P* for intercept=0.3859 and global test *P=*0.1520) were found in this MR analysis (**Tables S3**, **S4**), the leave-one-out analysis suggests that the results were s robust (*P*=0.1666) (**Figure S1A**).

For AD, there was no evidence of a potential causal association between SLE and AD risk (OR=1.0000, 95% CI: 0.9999-1.0001, *P*=0.7264). The findings of MR-Egger (OR=1.0000, 95% CI: 0.9998-1.0002, *P*=0.8934) and WM (OR=1.0000, 95% CI: 0.9998-1.0001, *P*=0.6230) were consistent (**Figure 4B**). In addition, Cochran’s Q test suggested no significant heterogeneity (*P*=0.1756) (**Tables S3**, **S4**). MR-Egger regression (*P* for intercept = 0.9628) and MR-PRESSO (global test *P=*0.2008) also did not find significant horizontal pleiotropy. Moreover, the leave-one-out test indicates that our results were stable (*P*=0.7264) (**Figure S1B**).

For VaD, we found that the three methods also reached different conclusions. IVW method had weak evidence of borderline significance for the causal genetic association between SLE and VaD risk (OR=0.9365, 95% CI: 0.8769-1.0002, *P*=0.0506). No such association was found using the MR-Egger method (OR=0.9292, 95% CI: 0.8043-1.0735, *P*=0.3246) (**Figure 4C**). However, the WM method revealed a significant genetic correlation between SLE and VaD risk (OR=0.8996, 95% CI: 0.8297-0.9754, *P*=0.0103). Since there was no significant heterogeneity (*P*=0.0850) or horizontal pleiotropy (*P* for intercept = 0.9048 and global test *P=*0.0966), we considered the result of IVW more credible (**Tables S3**, **S4**). The stability of the MR estimates was also verified by the leave-one-out test (**Figure S1C**).

For FTD, we did not find a genetic association with SLE (OR=1.0467, 95% CI: 0.9074-1.2075, *P*=0.5310). Similar results were shown on MR-Egger (OR=1.0721, 95% CI: 0.7085-1.6221, *P*=0.7487) and WM (OR=1.0969, 95% CI: 0.9126-1.3185, *P*=0.3243) (**Figure 4D**). The results of Cochran’s Q test, MR Egger regression, MR-PRESSO and the leave-one-out test showed that the MR estimates were relatively robust (**Tables S3**, **S4**) (**Figure S1D**).

For DLB, no genetically significant association was found with SLE (OR=1.0308,95% CI: 0.9829-1.0810, *P*=0.2112). MR-Egger (OR=0.9571,95% CI: 0.8650-1.0590, *P*=0.4016) and WM (OR=1.0201, 95% CI: 0.9550-1.0897, *P*=0.5536) revealed consistent conclusions (**Figure 4E**). Sensitivity analysis and heterogeneity test did not indicate potential horizontal pleiotropy and significant heterogeneity (**Tables S3**, **S4**). The leave-one-out test demonstrated that the MR estimate was stable when individual SNP was removed (**Figure S1E**). Finally, the funnel plots on SLE and dementia are presented in **Figure S2**.

## Discussion

In the present two-sample MR study, no genetic causal association was found between SLE and the risk of dementia.

Although cognitive impairment is one of the frequent clinical manifestations of NPSLE patients, progression to dementia is rare (39). A 5-year cohort study found that standardized neuropsychological test scores among patients with SLE were relatively stable and even found signs of improvement during the observation period (40). It revealed that cognitive impairment is stable and reversible in SLE patients. In addition, lupus activity did not appear to have a significant association with cognitive impairment (41).

In recent years, there has been increasing attention to the relationship between autoimmune diseases, especially SLE, and dementia. Several epidemiological studies have shown a potential association between SLE and dementia. Two nationwide population-based cohort studies found that SLE was associated with a higher risk of dementia (42, 43). Another large data analysis that included more than four thousand SLE patients and twenty-four thousand age and gender matched non-SLE controls found an increased risk of dementia in SLE patients (44). Recently, a meta-analysis by Zhao et al. integrating eleven relevant observational studies demonstrated that SLE adversely affects cognition and significantly increases dementia risk (14). In this study, only three relevant studies on the association between SLE and the risk of dementia were included and the variability among epidemiological studies regarding study design, methodology and quality, has made the association between SLE and dementia challenging to ascertain. Moreover, it is noteworthy that most of the current studies were observational. The evidence from observational studies should be interpreted with caution as it is unable to reveal causality and completely exclude the effects of confounding factors.

Our study found no direct genetic causality between SLE and dementia. The higher prevalence of dementia among SLE patients compared to the general population in the observational studies may be attributed to the following reasons. Firstly, pharmacological treatments might influence dementia risk in SLE patients to a certain extent. Glucocorticoids (GCs) play an essential role in the treatment of chronic inflammation (7), with their use in up to 80% of SLE patients, primarily for long courses of treatment. It has been well-established that GCs have neurotoxic effects (45–47). A cohort study that included 123 SLE patients with at least 3 years of follow-up found that long-term use of GCs was a predictor of cognitive impairment (45). The specific mechanism may be that long-term GCs use reduces the hippocampus volume, a crucial brain region in charge of learning and memory. In addition, high plasma levels of GCs and suppression of microglia glucocorticoid receptors (GRs) cause changes in microglia morphology and branching in the hippocampal region. These changes play an important role in the onset and progression of dementia (48, 49). Currently, though disease-modifying antirheumatic drugs (DMARDs) therapy may control rheumatic disease activity effectively, there is conflicting evidence for its effects on cognitive dysfunction. A case-control study that included 957 patients showed that conventional synthetic DMARDs (csDMARDs) (hydroxychloroquine, methotrexate, and sulfasalazine) commonly used in SLE were significantly associated with an increased dementia risk, whereas biological DMARDs (bDMARDs) were not (50). Another study found that bDMARDs, specifical etanercept, were shown to reduce the dementia risk significantly (51). Secondly, approximately 40% of patients with SLE are confirmed positive for anticardiolipin antibodies (aPL) and 50%-70% of these progress to secondary antiphospholipid syndrome (APS). Previous studies have observed a higher risk of dementia in SLE patients with secondary APS, which may be due to the hypercoagulable state and microembolism (52, 53). Our study did not find significant horizontal pleiotropy, therefore SLE patients with secondary APS may have been excluded. Thirdly, SLE is a multisystem autoimmune connective tissue disease with multiple co-morbidities. Approximately 51% of patients have three or more co-morbidities, such as hypertension, obesity, dyslipidemia, and depression. The presence of these diseases has been proven to be independent risk factors for dementia which may lead to an overestimation of the association between SLE and dementia risk (54, 55). Finally, SLE is a remarkably heterogeneous autoimmune disease and may exist different disease groups (56–58). Up to now, several studies have observed significant differences in pathogenesis, clinical manifestations, and genetic susceptibility among patients with SLE from different ancestral backgrounds (59–61). GWAS have attempted to partially explain the complex genetic structure of SLE. However, some alleles have not been sequenced in diverse ancestral backgrounds. Thus, the possibility remains that important causative genes may be buried.

To our knowledge, this is the first MR study to investigate the causal association between SLE and dementia risk. Our research has several strengths. Firstly, the main advantage is the MR design, which can avoid interference from confounding factors and reverse causal association. Secondly, we strictly screened SNPs using plink clumping to ensure the independence of IVs. Thirdly, the F-statistics of the included SNPs were all over 80, so the included genetic instruments were relatively powerful.

However, several limitations are worth mentioning. First, the sample size of the study is relatively small compared to population-based observational studies, although we use the largest and most recent GWAS database. Second, epigenetic issues such as DNA methylation, RNA editing and transposons inactive are the unavoidable shortcoming of MR analysis. Third, there might be an ethnic bias in our study due to all the selected GWAS database populations being of European ancestry. Forth, detailed demographic and clinical data on participants were not available, so the subgroup analysis was not performed.

## Conclusion

In summary, our findings do not support a causal association between SLE and dementia risk, which was inconsistent with previous observational studies. In the future, whole genome sequencing is needed for NPSLE patients to better explain genetic variation. Updated MR studies will be warranted to validate our results when more efficient methods are available to produce less biased MR estimates or when more extensive GWAS summary data are accessible. Meanwhile, further multicenter, large-sample, and follow-up studies should be conducted to longitudinal assess the patient’s cognitive function, dynamic monitor laboratory indicators and imaging changes to identify predictive and prognostic factors in the real world.

## Data availability statement

The original contributions presented in the study are included in the article/**Supplementary Material**. Further inquiries can be directed to the corresponding authors.

## Ethics statement

Ethical approval was not provided for this study on human participants because we used the publicly available GWAS catalog to conduct a two-sample MR study. No additional ethical approval was required due to the re-analysis of previously summary-level data. The patients/participants provided their written informed consent to participate in this study.

## Author contributions

TJ: present idea, perform MR analysis and manuscript writing. WH: evaluate the quality of MR and manuscript writing. FC: Search of the database and quality assessment. XY: figure and table drawing. SG: assisted funding. ZY and CX: study supervision and final approvement. All authors contributed to the article and approved the submitted version.

## Supplementary material

Supplementary Figure 1 Leave-one-out plots of SLE and all dementia **(A)**, Alzheimer’s disease **(B)**, vascular dementia **(C)**, frontotemporal dementia **(D)**, dementia with lewy body **(E)**. The leave-one-out plot visualizes how the causal estimates (point with horizontal line) for the effect of SLE on dementia are influenced by the exclusion of individual SNPs. The leave-one-out analysis suggests no individual SNP significantly affect the risk of SLE on dementia, which indicates that the results are reliable.

Supplementary Figure 2 Funnel plot on SLE and all dementia **(A)**, Alzheimer’s disease **(B)**, vascular dementia **(C)**, frontotemporal dementia **(D)**, dementia with lewy body **(E)**. The funnel plots are symmetric, which shows that the absence of polymorphism.
