# Supplementary material for: Causal association between systemic lupus erythematosus and the risk of dementia: A Mendelian randomization study
Source: Front Immunol. 2022 Dec 8;13:1063110. doi: 10.3389/fimmu.2022.1063110 (PMC9773372; doi:10.3389/fimmu.2022.1063110)
Supplement: Supplementary file 1 [file DataSheet_1.docx]

**Table S1** Characteristics of instrumental variables for Systemic Lupus Erythematosus

|  | **SNP** | **EA** | **OA** | **Gene** | **Samplesize** | **SE** | **β** | **EA** | **OA** | **EAF** | ***P* value** | **R^2^** | **F - statistic** |
| --- | --- | --- | --- | --- | --- | --- | --- | --- | --- | --- | --- | --- | --- |
| 1 | rs6679677 | A | C | PHTF1 | 14267 | 0.0465 | 0.3365 | A | C | 0.0915 | 4.55E-13 | 0.0188 | 273.6516 |
| 2 | rs4661543 | G | T | KAZN | 14267 | 0.0424 | 0.2744 | G | T | 0.8728 | 9.40E-11 | 0.0167 | 242.6125 |
| 3 | rs10912578 | G | A | snoU13 | 14267 | 0.0310 | -0.2469 | G | A | 0.3012 | 1.65E-15 | 0.0257 | 375.5757 |
| 4 | rs17849501 | T | C | NCF2 | 14267 | 0.0499 | 0.8109 | T | C | 0.9404 | 1.81E-59 | 0.0737 | 1135.2271 |
| 5 | rs6671847 | A | G | FCGR2A | 14267 | 0.0290 | 0.1989 | A | G | 0.4871 | 6.64E-12 | 0.0198 | 287.5244 |
| 6 | rs4916215 | T | C | RP11-296O14.1 | 14267 | 0.0340 | 0.2231 | T | C | 0.2545 | 5.07E-11 | 0.0189 | 274.7210 |
| 7 | rs12094036 | C | T | NCF2 | 14267 | 0.0579 | -0.3285 | C | T | 0.0815 | 1.37E-08 | 0.0162 | 234.2577 |
| 8 | rs13019891 | T | G | IL1F10 | 14267 | 0.0290 | -0.5621 | T | G | 0.5487 | 1.65E-83 | 0.1565 | 2646.4787 |
| 9 | rs2573219 | C | A | ECEL1P1 | 14267 | 0.0429 | 0.5878 | C | A | 0.0865 | 1.13E-42 | 0.0546 | 823.8552 |
| 10 | rs10200680 | T | C | KCNE4 | 14267 | 0.0425 | -0.2485 | T | C | 0.8559 | 4.96E-09 | 0.0152 | 220.5817 |
| 11 | rs268124 | T | C | SPRED2 | 14267 | 0.0324 | 0.1863 | T | C | 0.2744 | 8.60E-09 | 0.0138 | 199.9838 |
| 12 | rs2459611 | T | C | STAT4 | 14267 | 0.0452 | 0.2614 | T | C | 0.1252 | 7.62E-09 | 0.0150 | 216.6993 |
| 13 | rs4274624 | T | C | STAT4 | 14267 | 0.0327 | -0.559`6 | T | C | 0.2316 | 9.73E-66 | 0.1115 | 1789.5057 |
| 14 | rs10048743 | T | G | IKZF2 | 14267 | 0.0412 | -0.2311 | T | G | 0.1412 | 2.04E-08 | 0.0130 | 187.2130 |
| 15 | rs34703115 | C | T | SLC8A1-AS1 | 14267 | 0.1048 | -0.6162 | C | T | 0.0328 | 4.08E-09 | 0.0241 | 352.1324 |
| 16 | rs1464446 | T | G | SIAH2 | 14267 | 0.0401 | -0.3285 | T | G | 0.1789 | 2.79E-16 | 0.0317 | 467.0695 |
| 17 | rs9852014 | G | A | MRPL3 | 14267 | 0.0493 | 0.6206 | G | A | 0.9254 | 2.26E-36 | 0.0532 | 801.1074 |
| 18 | rs13136219 | T | C | BANK1 | 14267 | 0.0278 | -0.1744 | T | C | 0.6203 | 3.50E-10 | 0.0143 | 207.2368 |
| 19 | rs1078324 | A | C | PPARGC1B | 14267 | 0.0782 | -0.7134 | A | C | 0.0497 | 7.11E-20 | 0.0481 | 720.3078 |
| 20 | rs4388254 | T | C | CTB-1I21.1 | 14267 | 0.0604 | 0.3784 | T | C | 0.9294 | 3.71E-10 | 0.0188 | 273.2335 |
| 21 | rs2431697 | C | T | NCF2 | 14267 | 0.0293 | -0.2231 | C | T | 0.9404 | 2.60E-14 | 0.0056 | 80.0685 |
| 22 | rs6889239 | C | T | TNIP1 | 14267 | 0.0317 | 0.2776 | C | T | 0.2575 | 2.19E-18 | 0.0295 | 433.2189 |
| 23 | rs389884 | G | A | STK19 | 14267 | 0.0432 | 0.9282 | G | A | 0.0736 | 2.92E-102 | 0.1175 | 1899.1543 |
| 24 | rs9274357 | T | C | HLA-DQB1 | 14267 | 0.0352 | 0.4574 | T | C | 0.7763 | 1.28E-38 | 0.0727 | 1117.9017 |
| 25 | rs7768653 | T | C | ATG5 | 14267 | 0.0297 | -0.2070 | T | C | 0.4016 | 3.11E-12 | 0.0206 | 300.0028 |
| 26 | rs12524498 | T | G | HCP5 | 14267 | 0.1208 | -0.6733 | T | G | 0.9891 | 2.48E-08 | 0.0098 | 140.8349 |
| 27 | rs58721818 | T | C | AL356739.1 | 14267 | 0.0756 | 0.6575 | T | C | 0.9751 | 3.38E-18 | 0.0210 | 305.9024 |
| 28 | rs150180633 | T | C | TBC1D22B | 14267 | 0.0690 | 0.9282 | T | C | 0.9831 | 2.66E-41 | 0.0286 | 420.4382 |
| 29 | rs28361029 | A | G | GRCh37 | 14267 | 0.0614 | -0.3857 | A | G | 0.0101 | 3.27E-10 | 0.0030 | 42.5522 |
| 30 | rs35000415 | T | C | IRF5 | 14267 | 0.0415 | 0.5878 | T | C | 0.8996 | 1.86E-45 | 0.0624 | 949.5369 |
| 31 | rs2736332 | C | G | FAM167A | 14267 | 0.0321 | 0.2776 | C | G | 0.2694 | 4.83E-18 | 0.0303 | 446.3747 |
| 32 | rs7823055 | T | G | RP1 | 14267 | 0.0286 | -0.3507 | T | G | 0.4235 | 1.64E-34 | 0.0600 | 911.1935 |
| 33 | rs7899626 | T | C | ARID5B | 14267 | 0.0333 | 0.1823 | T | C | 0.6372 | 4.19E-08 | 0.0154 | 222.6638 |
| 34 | rs7097397 | A | G | WDFY4 | 14267 | 0.0287 | -0.1863 | A | G | 0.3956 | 8.60E-11 | 0.0166 | 240.8347 |
| 35 | rs58688157 | G | A | CDHR5 | 14267 | 0.0336 | -0.2231 | G | A | 0.7316 | 2.97E-11 | 0.0196 | 284.5150 |
| 36 | rs353608 | G | A | AL356215.1 | 14267 | 0.0280 | 0.1863 | G | A | 0.5477 | 2.93E-11 | 0.0172 | 249.6733 |
| 37 | rs73050535 | T | C | RP11-429A20.3 | 14267 | 0.1241 | -0.7134 | T | C | 0.9702 | 9.11E-09 | 0.0294 | 432.4694 |
| 38 | rs597808 | G | A | ATXN2 | 14267 | 0.0295 | -0.1625 | G | A | 0.5338 | 3.51E-08 | 0.0131 | 190.0238 |
| 39 | rs1143679 | A | G | ITGAM | 14267 | 0.0400 | 0.5822 | A | G | 0.1312 | 5.03E-48 | 0.0773 | 1194.6825 |
| 40 | rs28834423 | C | G | ITGAM | 14267 | 0.0365 | 0.4574 | C | G | 0.1829 | 5.65E-36 | 0.0625 | 951.6512 |
| 41 | rs13332649 | G | A | LINC02132 | 14267 | 0.0376 | -0.3147 | G | A | 0.8022 | 5.43E-17 | 0.0314 | 462.9181 |
| 42 | rs143123127 | A | G | IKZF3 | 14267 | 0.0840 | 0.4700 | A | G | 0.0308 | 2.23E-08 | 0.0132 | 190.6491 |
| 43 | rs35251378 | A | G | TYK2 | 14267 | 0.0324 | -0.2357 | A | G | 0.2694 | 3.61E-13 | 0.0219 | 318.9951 |
| 44 | rs73068668 | A | G | PPP6R1 | 14267 | 0.0575 | -0.3147 | A | G | 0.0954 | 4.40E-08 | 0.0171 | 248.0955 |
| 45 | rs3747093 | A | G | CCDC116 | 14267 | 0.0345 | 0.2624 | A | G | 0.2018 | 2.88E-14 | 0.0222 | 323.5059 |

SNP:single nucleotide polymorphisms; EA: effect allele; OA: other allele; EAF: effect allele frequency; SE, standard error

**Table S2.1** SNPs from GWAS on Systemic Lupus Erythematosus and any dementia

|  | | | **Exposure (Systemic Lupus Erythematosus)** | | |  | **Outcome (Any dementia)** | | | | |
| --- | --- | --- | --- | --- | --- | --- | --- | --- | --- | --- | --- |
| **SNP** | **EA** | **OA** | **β** | **SE** | ***P* value** |  | **Case** | **Control** | **β** | **SE** | ***P* value** |
| rs10048743 | T | G | -0.2311 | 0.0412 | 2.04E-08 |  | 7,284 | 209,487 | 0.0005 | 0.0249 | 0.9840 |
| rs10200680 | T | C | -0.2485 | 0.0425 | 4.96E-09 |  | 7,284 | 209,487 | 0.0067 | 0.0255 | 0.7928 |
| rs1078324 | A | C | -0.7134 | 0.0782 | 7.11E-20 |  | 7,284 | 209,487 | 0.0236 | 0.0482 | 0.6246 |
| rs10912578 | G | A | -0.2469 | 0.0310 | 1.65E-15 |  | 7,284 | 209,487 | -0.0004 | 0.0188 | 0.9830 |
| rs1143679 | A | G | 0.5822 | 0.0400 | 5.03E-48 |  | 7,284 | 209,487 | -0.0263 | 0.0297 | 0.3754 |
| rs12094036 | C | T | -0.3285 | 0.0579 | 1.37E-08 |  | 7,284 | 209,487 | 0.0047 | 0.0328 | 0.8860 |
| rs12524498 | T | G | -0.6733 | 0.1208 | 2.48E-08 |  | 7,284 | 209,487 | -0.0290 | 0.0678 | 0.6687 |
| rs13019891 | T | G | -0.5621 | 0.0290 | 1.65E-83 |  | 7,284 | 209,487 | 0.0060 | 0.0193 | 0.7559 |
| rs13136219 | T | C | -0.1744 | 0.0278 | 3.50E-10 |  | 7,284 | 209,487 | 0.0450 | 0.0198 | 0.0232 |
| rs13332649 | G | A | -0.3147 | 0.0376 | 5.43E-17 |  | 7,284 | 209,487 | -0.0012 | 0.0217 | 0.9558 |
| rs143123127 | A | G | 0.4700 | 0.0840 | 2.23E-08 |  | 7,284 | 209,487 | -0.0844 | 0.0479 | 0.0782 |
| rs1464446 | T | G | -0.3285 | 0.0401 | 2.79E-16 |  | 7,284 | 209,487 | -0.0172 | 0.0250 | 0.4914 |
| rs150180633 | T | C | 0.9282 | 0.0690 | 2.66E-41 |  | 7,284 | 209,487 | 0.0481 | 0.1552 | 0.7566 |
| rs17849501 | T | C | 0.8109 | 0.0499 | 1.81E-59 |  | 7,284 | 209,487 | -0.0398 | 0.0502 | 0.4283 |
| rs2431697 | C | T | -0.2231 | 0.0293 | 2.60E-14 |  | 7,284 | 209,487 | 0.0538 | 0.0196 | 0.0061 |
| rs2459611 | T | C | 0.2614 | 0.0452 | 7.62E-09 |  | 7,284 | 209,487 | -0.0080 | 0.0423 | 0.8501 |
| rs2573219 | C | A | 0.5878 | 0.0429 | 1.13E-42 |  | 7,284 | 209,487 | -0.0381 | 0.0343 | 0.2673 |
| rs268124 | T | C | 0.1863 | 0.0324 | 8.60E-09 |  | 7,284 | 209,487 | 0.0101 | 0.0198 | 0.6099 |
| rs2736332 | C | G | 0.2776 | 0.0321 | 4.83E-18 |  | 7,284 | 209,487 | 0.0127 | 0.0247 | 0.6070 |
| rs28361029 | A | G | -0.3857 | 0.0614 | 3.27E-10 |  | 7,284 | 209,487 | -0.0599 | 0.0441 | 0.1743 |
| rs28834423 | C | G | 0.4574 | 0.0365 | 5.65E-36 |  | 7,284 | 209,487 | -0.0312 | 0.0284 | 0.2716 |
| rs34703115 | C | T | -0.6162 | 0.1048 | 4.08E-09 |  | 7,284 | 209,487 | 0.0295 | 0.0537 | 0.5826 |
| rs35000415 | T | C | 0.5878 | 0.0415 | 1.86E-45 |  | 7,284 | 209,487 | -0.0025 | 0.0270 | 0.9263 |
| rs35251378 | A | G | -0.2357 | 0.0324 | 3.61E-13 |  | 7,284 | 209,487 | 0.0054 | 0.0217 | 0.8031 |
| rs353608 | G | A | 0.1863 | 0.0280 | 2.93E-11 |  | 7,284 | 209,487 | -0.0112 | 0.0191 | 0.5568 |
| rs3747093 | A | G | 0.2624 | 0.0345 | 2.88E-14 |  | 7,284 | 209,487 | 0.0014 | 0.0208 | 0.9464 |
| rs389884 | G | A | 0.9282 | 0.0432 | 2.92E-102 |  | 7,284 | 209,487 | -0.0270 | 0.0367 | 0.4623 |
| rs4274624 | T | C | -0.5596 | 0.0327 | 9.73E-66 |  | 7,284 | 209,487 | 0.0282 | 0.0227 | 0.2148 |
| rs4388254 | T | C | 0.3784 | 0.0604 | 3.71E-10 |  | 7,284 | 209,487 | 0.0077 | 0.0301 | 0.7984 |
| rs4661543 | G | T | 0.2744 | 0.0424 | 9.40E-11 |  | 7,284 | 209,487 | 0.0103 | 0.0390 | 0.7918 |
| rs4916215 | T | C | 0.2231 | 0.0340 | 5.07E-11 |  | 7,284 | 209,487 | 0.0103 | 0.0247 | 0.6772 |
| rs58688157 | G | A | -0.2231 | 0.0336 | 2.97E-11 |  | 7,284 | 209,487 | -0.0098 | 0.0237 | 0.6797 |
| rs58721818 | T | C | 0.6575 | 0.0756 | 3.38E-18 |  | 7,284 | 209,487 | -0.1103 | 0.0718 | 0.1244 |
| rs597808 | G | A | -0.1625 | 0.0295 | 3.51E-08 |  | 7,284 | 209,487 | -0.0076 | 0.0193 | 0.6931 |
| rs6671847 | A | G | 0.1989 | 0.0290 | 6.64E-12 |  | 7,284 | 209,487 | -0.0069 | 0.0194 | 0.7218 |
| rs6679677 | A | C | 0.3365 | 0.0465 | 4.55E-13 |  | 7,284 | 209,487 | 0.0407 | 0.0272 | 0.1341 |
| rs6889239 | C | T | 0.2776 | 0.0317 | 2.19E-18 |  | 7,284 | 209,487 | 0.0335 | 0.0213 | 0.1154 |
| rs7097397 | A | G | -0.1863 | 0.0287 | 8.60E-11 |  | 7,284 | 209,487 | 0.0030 | 0.0201 | 0.8813 |
| rs73050535 | T | C | -0.7134 | 0.1241 | 9.11E-09 |  | 7,284 | 209,487 | 0.1454 | 0.1404 | 0.3003 |
| rs73068668 | A | G | -0.3147 | 0.0575 | 4.40E-08 |  | 7,284 | 209,487 | -0.0346 | 0.0347 | 0.3184 |
| rs7768653 | T | C | -0.2070 | 0.0297 | 3.11E-12 |  | 7,284 | 209,487 | 0.0108 | 0.0192 | 0.5747 |
| rs7823055 | T | G | -0.3507 | 0.0286 | 1.64E-34 |  | 7,284 | 209,487 | 0.0021 | 0.0190 | 0.9119 |
| rs7899626 | T | C | 0.1823 | 0.0333 | 4.19E-08 |  | 7,284 | 209,487 | 0.0391 | 0.0197 | 0.0474 |
| rs9852014 | G | A | 0.6206 | 0.0493 | 2.26E-36 |  | 7,284 | 209,487 | -0.0008 | 0.0336 | 0.9810 |

SNP:single nucleotide polymorphisms; EA: effect allele; OA: other allele; SE, standard error

**Table S2.2** SNPs from GWAS on Systemic Lupus Erythematosus and Alzheimer’s disease

|  | | | **Exposure (Systemic Lupus Erythematosus)** | | |  | **Outcome (Alzheimer's disease)** | | | | |
| --- | --- | --- | --- | --- | --- | --- | --- | --- | --- | --- | --- |
| **SNP** | **EA** | **OA** | **β** | **SE** | ***P* value** |  | **Case** | **Control** | **β** | **SE** | ***P* value** |
| rs10048743 | T | G | -0.23111 | 0.04121 | 2.04E-08 |  | 954 | 487,331 | -0.00001 | 0.00014 | 0.92 |
| rs10200680 | T | C | -0.24846 | 0.04248 | 4.96E-09 |  | 954 | 487,331 | 0.00019 | 0.00013 | 0.15 |
| rs1078324 | A | C | -0.71335 | 0.07817 | 7.11E-20 |  | 954 | 487,331 | -0.00009 | 0.00021 | 0.65 |
| rs10912578 | G | A | -0.24686 | 0.03099 | 1.65E-15 |  | 954 | 487,331 | 0.00016 | 0.00010 | 0.10 |
| rs1143679 | A | G | 0.58222 | 0.03999 | 5.03E-48 |  | 954 | 487,331 | 0.00005 | 0.00016 | 0.77 |
| rs12094036 | C | T | -0.32850 | 0.05786 | 1.37E-08 |  | 954 | 487,331 | 0.00027 | 0.00017 | 0.11 |
| rs12524498 | T | G | -0.67335 | 0.12079 | 2.48E-08 |  | 954 | 487,331 | 0.00005 | 0.00034 | 0.89 |
| rs13019891 | T | G | -0.56212 | 0.02903 | 1.65E-83 |  | 954 | 487,331 | -0.00006 | 0.00009 | 0.51 |
| rs13136219 | T | C | -0.17435 | 0.02779 | 3.50E-10 |  | 954 | 487,331 | 0.00012 | 0.00010 | 0.21 |
| rs13332649 | G | A | -0.31471 | 0.03757 | 5.43E-17 |  | 954 | 487,331 | 0.00002 | 0.00012 | 0.88 |
| rs143123127 | A | G | 0.47000 | 0.08403 | 2.23E-08 |  | 954 | 487,331 | -0.00021 | 0.00026 | 0.43 |
| rs1464446 | T | G | -0.32850 | 0.04015 | 2.79E-16 |  | 954 | 487,331 | -0.00015 | 0.00012 | 0.21 |
| rs150180633 | T | C | 0.92822 | 0.06896 | 2.66E-41 |  | 954 | 487,331 | -0.00004 | 0.00037 | 0.92 |
| rs17849501 | T | C | 0.81093 | 0.04986 | 1.81E-59 |  | 954 | 487,331 | -0.00006 | 0.00021 | 0.78 |
| rs2459611 | T | C | 0.26137 | 0.04525 | 7.62E-09 |  | 954 | 487,331 | -0.00009 | 0.00016 | 0.55 |
| rs2573219 | C | A | 0.58779 | 0.04293 | 1.13E-42 |  | 954 | 487,331 | -0.00007 | 0.00016 | 0.66 |
| rs268124 | T | C | 0.18633 | 0.03237 | 8.60E-09 |  | 954 | 487,331 | -0.00008 | 0.00011 | 0.42 |
| rs2736332 | C | G | 0.27763 | 0.03207 | 4.83E-18 |  | 954 | 487,331 | -0.00013 | 0.00011 | 0.22 |
| rs28834423 | C | G | 0.45743 | 0.03653 | 5.65E-36 |  | 954 | 487,331 | -0.00015 | 0.00013 | 0.26 |
| rs34703115 | C | T | -0.61619 | 0.10478 | 4.08E-09 |  | 954 | 487,331 | 0.00057 | 0.00029 | 0.05 |
| rs35000415 | T | C | 0.58779 | 0.04154 | 1.86E-45 |  | 954 | 487,331 | 0.00002 | 0.00017 | 0.89 |
| rs35251378 | A | G | -0.23572 | 0.03243 | 3.61E-13 |  | 954 | 487,331 | 0.00003 | 0.00010 | 0.78 |
| rs353608 | G | A | 0.18633 | 0.02802 | 2.93E-11 |  | 954 | 487,331 | 0.00009 | 0.00009 | 0.34 |
| rs3747093 | A | G | 0.26236 | 0.03451 | 2.88E-14 |  | 954 | 487,331 | 0.00010 | 0.00012 | 0.40 |
| rs389884 | G | A | 0.92822 | 0.04323 | 2.92E-102 |  | 954 | 487,331 | -0.00010 | 0.00014 | 0.48 |
| rs4274624 | T | C | -0.55962 | 0.03268 | 9.73E-66 |  | 954 | 487,331 | 0.00002 | 0.00011 | 0.84 |
| rs4388254 | T | C | 0.37844 | 0.06040 | 3.71E-10 |  | 954 | 487,331 | 0.00025 | 0.00025 | 0.31 |
| rs4661543 | G | T | 0.27444 | 0.04238 | 9.40E-11 |  | 954 | 487,331 | 0.00032 | 0.00014 | 0.02 |
| rs4916215 | T | C | 0.22314 | 0.03397 | 5.07E-11 |  | 954 | 487,331 | 0.00015 | 0.00010 | 0.16 |
| rs58688157 | G | A | -0.22314 | 0.03356 | 2.97E-11 |  | 954 | 487,331 | -0.00016 | 0.00010 | 0.12 |
| rs58721818 | T | C | 0.65752 | 0.07559 | 3.38E-18 |  | 954 | 487,331 | -0.00023 | 0.00027 | 0.38 |
| rs597808 | G | A | -0.16252 | 0.02947 | 3.51E-08 |  | 954 | 487,331 | 0.00000 | 0.00004 | 0.99 |
| rs6671847 | A | G | 0.19885 | 0.02897 | 6.64E-12 |  | 954 | 487,331 | -0.00013 | 0.00009 | 0.15 |
| rs6679677 | A | C | 0.33647 | 0.04649 | 4.55E-13 |  | 954 | 487,331 | 0.00025 | 0.00015 | 0.11 |
| rs6889239 | C | T | 0.27763 | 0.03174 | 2.19E-18 |  | 954 | 487,331 | -0.00017 | 0.00011 | 0.13 |
| rs7097397 | A | G | -0.18633 | 0.02871 | 8.60E-11 |  | 954 | 487,331 | 0.00001 | 0.00012 | 0.95 |
| rs73050535 | T | C | -0.71335 | 0.12413 | 9.11E-09 |  | 954 | 487,331 | -0.00072 | 0.00030 | 0.02 |
| rs73068668 | A | G | -0.31471 | 0.05749 | 4.40E-08 |  | 954 | 487,331 | -0.00021 | 0.00018 | 0.24 |
| rs7768653 | T | C | -0.20701 | 0.02969 | 3.11E-12 |  | 954 | 487,331 | 0.00008 | 0.00009 | 0.42 |
| rs7823055 | T | G | -0.35066 | 0.02862 | 1.64E-34 |  | 954 | 487,331 | 0.00009 | 0.00009 | 0.32 |
| rs7899626 | T | C | 0.18232 | 0.03325 | 4.19E-08 |  | 954 | 487,331 | 0.00009 | 0.00010 | 0.39 |
| rs9852014 | G | A | 0.62058 | 0.04927 | 2.26E-36 |  | 954 | 487,331 | -0.00010 | 0.00018 | 0.57 |

SNP:single nucleotide polymorphisms; EA: effect allele; OA: other allele; SE, standard error

**Table S2.3** SNPs from GWAS on Systemic Lupus Erythematosus and Vascular dementia

|  | | | **Exposure (Systemic Lupus Erythematosus)** | | |  | **Outcome (Vascular dementia)** | | | | |
| --- | --- | --- | --- | --- | --- | --- | --- | --- | --- | --- | --- |
| **SNP** | **EA** | **OA** | **β** | **SE** | ***P* value** |  | **Case** | **Control** | **β** | **SE** | ***P* value** |
| rs10048743 | T | G | -0.2311 | 0.0412 | 2.04E-08 |  | 881 | 211,508 | -0.0272 | 0.0636 | 0.67 |
| rs10200680 | T | C | -0.2485 | 0.0425 | 4.96E-09 |  | 881 | 211,508 | 0.0301 | 0.0660 | 0.65 |
| rs1078324 | A | C | -0.7134 | 0.0782 | 7.11E-20 |  | 881 | 211,508 | -0.0025 | 0.1254 | 0.98 |
| rs10912578 | G | A | -0.2469 | 0.0310 | 1.65E-15 |  | 881 | 211,508 | 0.0449 | 0.0529 | 0.40 |
| rs1143679 | A | G | 0.5822 | 0.0400 | 5.03E-48 |  | 881 | 211,508 | -0.0802 | 0.0762 | 0.29 |
| rs12094036 | C | T | -0.3285 | 0.0579 | 1.37E-08 |  | 881 | 211,508 | 0.0338 | 0.0848 | 0.69 |
| rs12524498 | T | G | -0.6733 | 0.1208 | 2.48E-08 |  | 881 | 211,508 | 0.5174 | 0.1727 | 0.00 |
| rs13019891 | T | G | -0.5621 | 0.0290 | 1.65E-83 |  | 881 | 211,508 | 0.0719 | 0.0494 | 0.15 |
| rs13136219 | T | C | -0.1744 | 0.0278 | 3.50E-10 |  | 881 | 211,508 | 0.1478 | 0.0511 | 0.00 |
| rs13332649 | G | A | -0.3147 | 0.0376 | 5.43E-17 |  | 881 | 211,508 | -0.0074 | 0.0555 | 0.89 |
| rs143123127 | A | G | 0.4700 | 0.0840 | 2.23E-08 |  | 881 | 211,508 | -0.2683 | 0.1223 | 0.03 |
| rs1464446 | T | G | -0.3285 | 0.0401 | 2.79E-16 |  | 881 | 211,508 | 0.0430 | 0.0643 | 0.50 |
| rs150180633 | T | C | 0.9282 | 0.0690 | 2.66E-41 |  | 881 | 211,508 | -0.0065 | 0.3899 | 0.99 |
| rs17849501 | T | C | 0.8109 | 0.0499 | 1.81E-59 |  | 881 | 211,508 | -0.0821 | 0.1282 | 0.52 |
| rs2431697 | C | T | -0.2231 | 0.0293 | 2.60E-14 |  | 881 | 211,508 | 0.0783 | 0.0504 | 0.12 |
| rs2459611 | T | C | 0.2614 | 0.0452 | 7.62E-09 |  | 881 | 211,508 | -0.2407 | 0.1077 | 0.03 |
| rs2573219 | C | A | 0.5878 | 0.0429 | 1.13E-42 |  | 881 | 211,508 | -0.0050 | 0.0872 | 0.95 |
| rs268124 | T | C | 0.1863 | 0.0324 | 8.60E-09 |  | 881 | 211,508 | 0.0961 | 0.0508 | 0.06 |
| rs2736332 | C | G | 0.2776 | 0.0321 | 4.83E-18 |  | 881 | 211,508 | 0.1323 | 0.0640 | 0.04 |
| rs28361029 | A | G | -0.3857 | 0.0614 | 3.27E-10 |  | 881 | 211,508 | 0.1217 | 0.1129 | 0.28 |
| rs28834423 | C | G | 0.4574 | 0.0365 | 5.65E-36 |  | 881 | 211,508 | -0.0760 | 0.0729 | 0.30 |
| rs34703115 | C | T | -0.6162 | 0.1048 | 4.08E-09 |  | 881 | 211,508 | 0.0318 | 0.1373 | 0.82 |
| rs35000415 | T | C | 0.5878 | 0.0415 | 1.86E-45 |  | 881 | 211,508 | 0.0407 | 0.0677 | 0.55 |
| rs35251378 | A | G | -0.2357 | 0.0324 | 3.61E-13 |  | 881 | 211,508 | 0.0338 | 0.0557 | 0.54 |
| rs353608 | G | A | 0.1863 | 0.0280 | 2.93E-11 |  | 881 | 211,508 | 0.0470 | 0.0492 | 0.34 |
| rs3747093 | A | G | 0.2624 | 0.0345 | 2.88E-14 |  | 881 | 211,508 | -0.0554 | 0.0537 | 0.30 |
| rs389884 | G | A | 0.9282 | 0.0432 | 2.92E-102 |  | 881 | 211,508 | 0.0104 | 0.0944 | 0.91 |
| rs4274624 | T | C | -0.5596 | 0.0327 | 9.73E-66 |  | 881 | 211,508 | 0.0659 | 0.0585 | 0.26 |
| rs4388254 | T | C | 0.3784 | 0.0604 | 3.71E-10 |  | 881 | 211,508 | -0.0482 | 0.0782 | 0.54 |
| rs4661543 | G | T | 0.2744 | 0.0424 | 9.40E-11 |  | 881 | 211,508 | 0.1211 | 0.1005 | 0.23 |
| rs4916215 | T | C | 0.2231 | 0.0340 | 5.07E-11 |  | 881 | 211,508 | -0.0598 | 0.0630 | 0.34 |
| rs58688157 | G | A | -0.2231 | 0.0336 | 2.97E-11 |  | 881 | 211,508 | -0.0322 | 0.0608 | 0.60 |
| rs58721818 | T | C | 0.6575 | 0.0756 | 3.38E-18 |  | 881 | 211,508 | -0.1562 | 0.1821 | 0.39 |
| rs597808 | G | A | -0.1625 | 0.0295 | 3.51E-08 |  | 881 | 211,508 | -0.0127 | 0.0500 | 0.80 |
| rs6671847 | A | G | 0.1989 | 0.0290 | 6.64E-12 |  | 881 | 211,508 | -0.0569 | 0.0495 | 0.25 |
| rs6679677 | A | C | 0.3365 | 0.0465 | 4.55E-13 |  | 881 | 211,508 | 0.0502 | 0.0701 | 0.47 |
| rs6889239 | C | T | 0.2776 | 0.0317 | 2.19E-18 |  | 881 | 211,508 | 0.0677 | 0.0545 | 0.21 |
| rs7097397 | A | G | -0.1863 | 0.0287 | 8.60E-11 |  | 881 | 211,508 | 0.0390 | 0.0511 | 0.44 |
| rs73050535 | T | C | -0.7134 | 0.1241 | 9.11E-09 |  | 881 | 211,508 | -0.5055 | 0.3689 | 0.17 |
| rs73068668 | A | G | -0.3147 | 0.0575 | 4.40E-08 |  | 881 | 211,508 | -0.1022 | 0.0899 | 0.26 |
| rs7768653 | T | C | -0.2070 | 0.0297 | 3.11E-12 |  | 881 | 211,508 | 0.0229 | 0.0493 | 0.64 |
| rs7823055 | T | G | -0.3507 | 0.0286 | 1.64E-34 |  | 881 | 211,508 | 0.0373 | 0.0495 | 0.45 |
| rs7899626 | T | C | 0.1823 | 0.0333 | 4.19E-08 |  | 881 | 211,508 | 0.0888 | 0.0506 | 0.08 |
| rs9852014 | G | A | 0.6206 | 0.0493 | 2.26E-36 |  | 881 | 211,508 | 0.0598 | 0.0904 | 0.51 |

SNP:single nucleotide polymorphisms; EA: effect allele; OA: other allele; SE, standard error

**Table S2.4** SNPs from GWAS on Systemic Lupus Erythematosus and Frontotemporal dementia

|  | | | **Exposure (Systemic Lupus Erythematosus)** | | |  | **Outcome (Frontotemporal dementia)** | | | | |
| --- | --- | --- | --- | --- | --- | --- | --- | --- | --- | --- | --- |
| **SNP** | **EA** | **OA** | **β** | **SE** | ***P* value** |  | **Case** | **Control** | **β** | **SE** | ***P* value** |
| rs1143679 | A | G | 0.5822 | 0.0400 | 5.03E-48 |  | 515 | 2,509 | 0.1151 | 0.1032 | 0.26 |
| rs12094036 | C | T | -0.3285 | 0.0579 | 1.37E-08 |  | 515 | 2,509 | -0.2715 | 0.1408 | 0.05 |
| rs13332649 | G | A | -0.3147 | 0.0376 | 5.43E-17 |  | 515 | 2,509 | -0.0758 | 0.0842 | 0.37 |
| rs2459611 | T | C | 0.2614 | 0.0452 | 7.62E-09 |  | 515 | 2,509 | 0.0600 | 0.1141 | 0.60 |
| rs2573219 | C | A | 0.5878 | 0.0429 | 1.13E-42 |  | 515 | 2,509 | 0.0871 | 0.1128 | 0.44 |
| rs2736332 | C | G | 0.2776 | 0.0321 | 4.83E-18 |  | 515 | 2,509 | 0.0602 | 0.0787 | 0.44 |
| rs28834423 | C | G | 0.4574 | 0.0365 | 5.65E-36 |  | 515 | 2,509 | 0.0218 | 0.0906 | 0.81 |
| rs35000415 | T | C | 0.5878 | 0.0415 | 1.86E-45 |  | 515 | 2,509 | -0.1925 | 0.1155 | 0.10 |
| rs4274624 | T | C | -0.5596 | 0.0327 | 9.73E-66 |  | 515 | 2,509 | -0.0227 | 0.0812 | 0.78 |
| rs4388254 | T | C | 0.3784 | 0.0604 | 3.71E-10 |  | 515 | 2,509 | 0.0602 | 0.1742 | 0.73 |
| rs4661543 | G | T | 0.2744 | 0.0424 | 9.40E-11 |  | 515 | 2,509 | -0.1604 | 0.1002 | 0.11 |
| rs6679677 | A | C | 0.3365 | 0.0465 | 4.55E-13 |  | 515 | 2,509 | 0.0411 | 0.1161 | 0.72 |
| rs6889239 | C | T | 0.2776 | 0.0317 | 2.19E-18 |  | 515 | 2,509 | 0.0020 | 0.0638 | 0.98 |
| rs7768653 | T | C | -0.2070 | 0.0297 | 3.11E-12 |  | 515 | 2,509 | 0.0238 | 0.0705 | 0.74 |

SNP:single nucleotide polymorphisms; EA: effect allele; OA: other allele; SE, standard error

**Table S2.5** SNPs from GWAS on Systemic Lupus Erythematosus and Dementia with Lewy bodies

|  | | | **Exposure (Systemic Lupus Erythematosus)** | | |  | **Outcome (Dementia with Lewy bodies)** | | | | |
| --- | --- | --- | --- | --- | --- | --- | --- | --- | --- | --- | --- |
| **SNP** | **EA** | **OA** | **β** | **SE** | ***P* value** |  | **Case** | **Control** | **β** | **SE** | ***P* value** |
| rs10048743 | T | G | -0.2311 | 0.0412 | 2.04E-08 |  | 2,591 | 4,027 | -0.0460 | 0.0534 | 0.39 |
| rs10200680 | T | C | -0.2485 | 0.0425 | 4.96E-09 |  | 2,591 | 4,027 | -0.0045 | 0.0528 | 0.93 |
| rs1078324 | A | C | -0.7134 | 0.0782 | 7.11E-20 |  | 2,591 | 4,027 | 0.1124 | 0.0781 | 0.15 |
| rs10912578 | G | A | -0.2469 | 0.0310 | 1.65E-15 |  | 2,591 | 4,027 | -0.0009 | 0.0396 | 0.98 |
| rs1143679 | A | G | 0.5822 | 0.0400 | 5.03E-48 |  | 2,591 | 4,027 | 0.0463 | 0.0543 | 0.39 |
| rs12094036 | C | T | -0.3285 | 0.0579 | 1.37E-08 |  | 2,591 | 4,027 | 0.0448 | 0.0701 | 0.52 |
| rs12524498 | T | G | -0.6733 | 0.1208 | 2.48E-08 |  | 2,591 | 4,027 | -0.0532 | 0.1458 | 0.72 |
| rs13019891 | T | G | -0.5621 | 0.0290 | 1.65E-83 |  | 2,591 | 4,027 | -0.0114 | 0.0373 | 0.76 |
| rs13136219 | T | C | -0.1744 | 0.0278 | 3.50E-10 |  | 2,591 | 4,027 | -0.0087 | 0.0384 | 0.82 |
| rs143123127 | A | G | 0.4700 | 0.0840 | 2.23E-08 |  | 2,591 | 4,027 | -0.0277 | 0.1016 | 0.79 |
| rs1464446 | T | G | -0.3285 | 0.0401 | 2.79E-16 |  | 2,591 | 4,027 | 0.0096 | 0.0485 | 0.84 |
| rs150180633 | T | C | 0.9282 | 0.0690 | 2.66E-41 |  | 2,591 | 4,027 | 0.1307 | 0.1714 | 0.45 |
| rs17849501 | T | C | 0.8109 | 0.0499 | 1.81E-59 |  | 2,591 | 4,027 | -0.0336 | 0.0868 | 0.70 |
| rs2431697 | C | T | -0.2231 | 0.0293 | 2.60E-14 |  | 2,591 | 4,027 | -0.0313 | 0.0373 | 0.40 |
| rs2459611 | T | C | 0.2614 | 0.0452 | 7.62E-09 |  | 2,591 | 4,027 | -0.0468 | 0.0589 | 0.43 |
| rs2573219 | C | A | 0.5878 | 0.0429 | 1.13E-42 |  | 2,591 | 4,027 | -0.0278 | 0.0619 | 0.65 |
| rs268124 | T | C | 0.1863 | 0.0324 | 8.60E-09 |  | 2,591 | 4,027 | 0.0153 | 0.0425 | 0.72 |
| rs2736332 | C | G | 0.2776 | 0.0321 | 4.83E-18 |  | 2,591 | 4,027 | 0.0212 | 0.0418 | 0.61 |
| rs28834423 | C | G | 0.4574 | 0.0365 | 5.65E-36 |  | 2,591 | 4,027 | 0.0606 | 0.0491 | 0.22 |
| rs34703115 | C | T | -0.6162 | 0.1048 | 4.08E-09 |  | 2,591 | 4,027 | 0.0930 | 0.1052 | 0.38 |
| rs35000415 | T | C | 0.5878 | 0.0415 | 1.86E-45 |  | 2,591 | 4,027 | 0.0174 | 0.0594 | 0.77 |
| rs35251378 | A | G | -0.2357 | 0.0324 | 3.61E-13 |  | 2,591 | 4,027 | -0.1676 | 0.0420 | 0.00 |
| rs353608 | G | A | 0.1863 | 0.0280 | 2.93E-11 |  | 2,591 | 4,027 | 0.0731 | 0.0372 | 0.05 |
| rs3747093 | A | G | 0.2624 | 0.0345 | 2.88E-14 |  | 2,591 | 4,027 | 0.0531 | 0.0469 | 0.26 |
| rs4274624 | T | C | -0.5596 | 0.0327 | 9.73E-66 |  | 2,591 | 4,027 | 0.0231 | 0.0447 | 0.60 |
| rs4388254 | T | C | 0.3784 | 0.0604 | 3.71E-10 |  | 2,591 | 4,027 | -0.0339 | 0.0894 | 0.70 |
| rs4661543 | G | T | 0.2744 | 0.0424 | 9.40E-11 |  | 2,591 | 4,027 | -0.0686 | 0.0546 | 0.21 |
| rs4916215 | T | C | 0.2231 | 0.0340 | 5.07E-11 |  | 2,591 | 4,027 | 0.0412 | 0.0419 | 0.33 |
| rs58688157 | G | A | -0.2231 | 0.0336 | 2.97E-11 |  | 2,591 | 4,027 | -0.0021 | 0.0409 | 0.96 |
| rs58721818 | T | C | 0.6575 | 0.0756 | 3.38E-18 |  | 2,591 | 4,027 | 0.1472 | 0.1051 | 0.16 |
| rs597808 | G | A | -0.1625 | 0.0295 | 3.51E-08 |  | 2,591 | 4,027 | 0.0022 | 0.0374 | 0.95 |
| rs6671847 | A | G | 0.1989 | 0.0290 | 6.64E-12 |  | 2,591 | 4,027 | 0.0002 | 0.0364 | 1.00 |
| rs6889239 | C | T | 0.2776 | 0.0317 | 2.19E-18 |  | 2,591 | 4,027 | 0.0084 | 0.0427 | 0.84 |
| rs7097397 | A | G | -0.1863 | 0.0287 | 8.60E-11 |  | 2,591 | 4,027 | -0.0311 | 0.0380 | 0.41 |
| rs73068668 | A | G | -0.3147 | 0.0575 | 4.40E-08 |  | 2,591 | 4,027 | 0.0840 | 0.0689 | 0.22 |
| rs7768653 | T | C | -0.2070 | 0.0297 | 3.11E-12 |  | 2,591 | 4,027 | 0.0252 | 0.0378 | 0.51 |
| rs7823055 | T | G | -0.3507 | 0.0286 | 1.64E-34 |  | 2,591 | 4,027 | -0.0113 | 0.0381 | 0.77 |
| rs7899626 | T | C | 0.1823 | 0.0333 | 4.19E-08 |  | 2,591 | 4,027 | 0.0315 | 0.0402 | 0.43 |
| rs9852014 | G | A | 0.6206 | 0.0493 | 2.26E-36 |  | 2,591 | 4,027 | 0.1099 | 0.0682 | 0.11 |

SNP:single nucleotide polymorphisms; EA: effect allele; OA: other allele; SE, standard error

**Table S3** Heterogeneity of MR analysis for Systemic lupus erythematosus and dementia risk

| **Exposure** | **Outcome** | **Method** | **Q** | **Q_df** | **Q_P val** |
| --- | --- | --- | --- | --- | --- |
| SLE | Any Dementia | MR Egger | 36.05 | 40 | 0.65 |
| SLE | Any Dementia | Inverse variance weighted | 36.82 | 41 | 0.66 |
| SLE | Alzheimer's disease | MR Egger | 47.07 | 38 | 0.15 |
| SLE | Alzheimer's disease | Inverse variance weighted | 47.07 | 39 | 0.18 |
| SLE | Vascular dementia | MR Egger | 53.91 | 40 | 0.07 |
| SLE | Vascular dementia | Inverse variance weighted | 53.93 | 41 | 0.08 |
| SLE | Frontotemporal dementia | MR Egger | 11.98 | 10 | 0.29 |
| SLE | Frontotemporal dementia | Inverse variance weighted | 11.99 | 11 | 0.36 |
| SLE | Dementia with Lewy bodies | MR Egger | 35.25 | 35 | 0.46 |
| SLE | Dementia with Lewy bodies | Inverse variance weighted | 37.88 | 36 | 0.38 |

SLE: Systemic lupus erythematosus

**Table S4** The results of MR-Egger intercept analysis, MR-Pleiotropy residual sum and outlier methods for risk of systemic lupus erythematosus and dementia

| **Exposure** | **Outcome** | **Egger_intercept** | **SE** | ***P* value** | **MR_PRESSO Global *P* value** |
| --- | --- | --- | --- | --- | --- |
| SLE | Any dementia | 0.0075 | 0.0086 | 0.39 | 0.15 |
| SLE | Alzheimer's disease | 0.0000 | 0.0000 | 0.96 | 0.20 |
| SLE | Vascular dementia | 0.0031 | 0.0257 | 0.90 | 0.10 |
| SLE | Frontotemporal dementia | -0.0101 | 0.0833 | 0.91 | 0.40 |
| SLE | Dementia with Lewy bodies | 0.0284 | 0.0175 | 0.11 | 0.40 |
