# Supplementary figures and images for: Causal association between systemic lupus erythematosus and the risk of dementia: A Mendelian randomization study
Source: Front Immunol. 2022 Dec 8;13:1063110. doi: 10.3389/fimmu.2022.1063110 (PMC9773372; doi:10.3389/fimmu.2022.1063110)

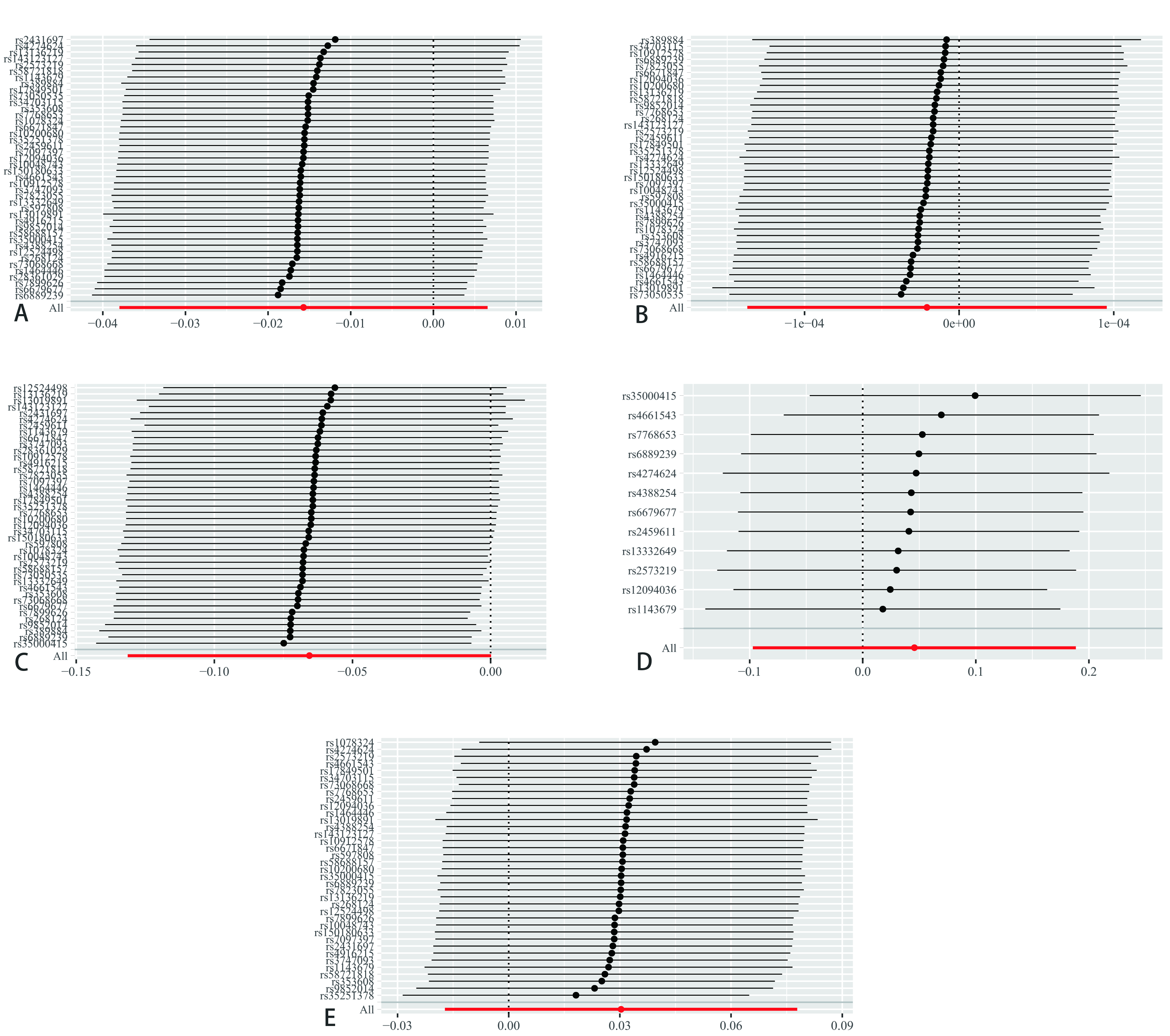

Supplement: Supplementary Figure 1 — Leave-one-out plots of SLE and all dementia (A), Alzheimer’s disease (B), vascular dementia (C), frontotemporal dementia (D), dementia with lewy body (E). The leave-one-out plot visualizes how the causal estimates (point with horizontal line) for the effect of SLE on dementia are influenced by the exclusion of individual SNPs. The leave-one-out analysis suggests no individual SNP significantly affect the risk of SLE on dementia, which indicates that the results are reliable. [file Image_1.tif]

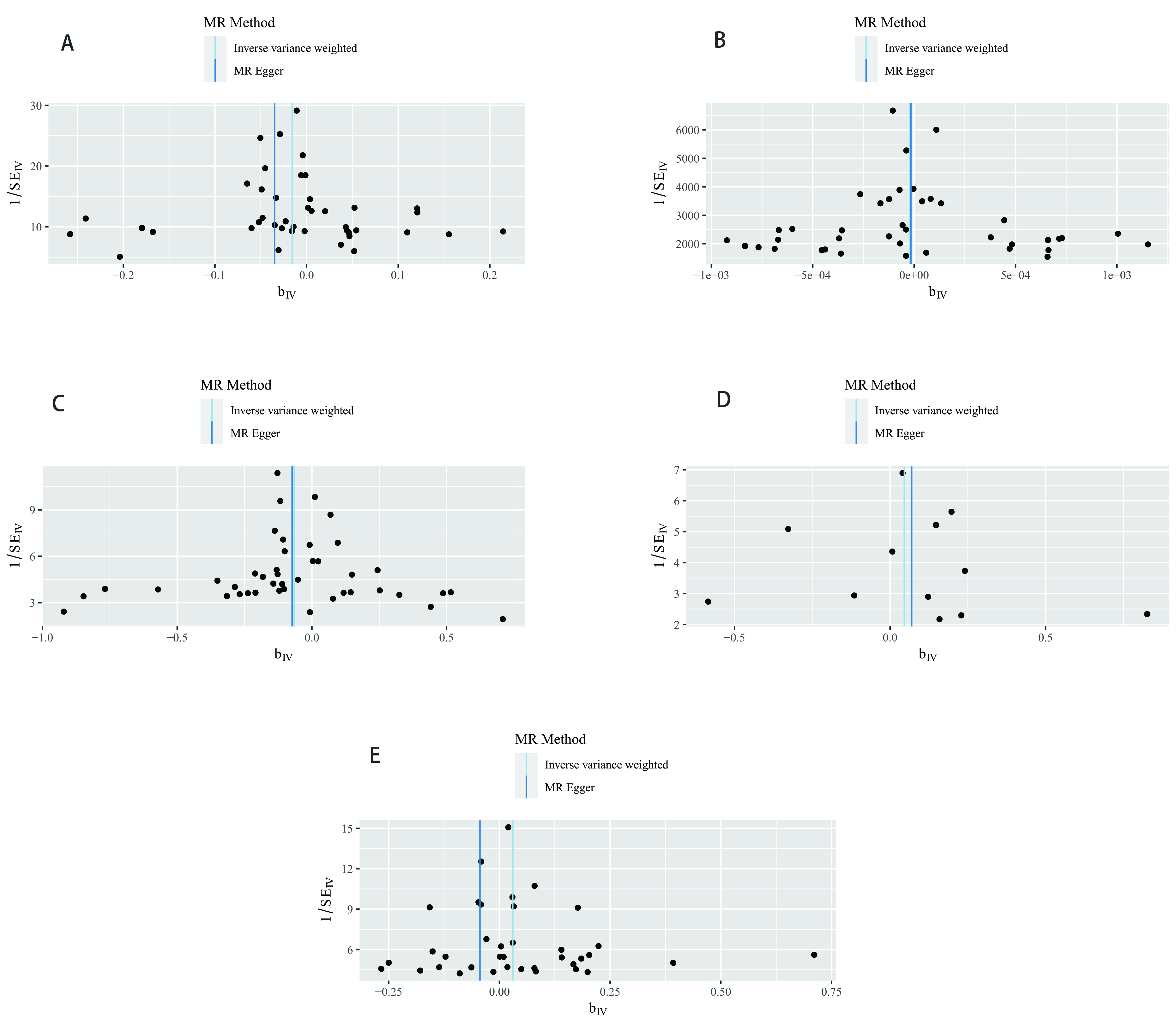

Supplement: Supplementary Figure 2 — Funnel plot on SLE and all dementia (A), Alzheimer’s disease (B), vascular dementia (C), frontotemporal dementia (D), dementia with lewy body (E). The funnel plots are symmetric, which shows that the absence of polymorphism. [file Image_2.tif]
